# New Insights into Infectious Skin and Mucosal Diseases: Translating Epidemiological Shifts into Tailored Clinical Practice

**DOI:** 10.3390/jcm15166184

**Published:** 2026-08-10

**Authors:** Giulia Ciccarese, Francesco Drago, Gloria Hoxhallari, Domenico Bonamonte, Aurora De Marco, Alexandre Raphael Meduri, Rossana Spadavecchia, Paolo Romita, Francesca Ambrogio, Caterina Foti

**Affiliations:** 1 Section of Dermatology, Department of Precision and Regenerative Medicine and Jonian Area, University of Bari “Aldo Moro”, Piazza Giulio Cesare, 11, 70124 Bari, Italy; domenico.bonamonte@uniba.it (D.B.); aurorademarco94@gmail.com (A.D.M.); meduri.alexandre@gmail.com (A.R.M.); rossana.spadavecchia@policlinico.ba.it (R.S.); paolo.romita@uniba.it (P.R.); dottambrogiofrancesca@gmail.com (F.A.); caterina.foti@uniba.it (C.F.); 2 Casa di Cura Villa Montallegro, 16145 Genoa, Italy; francescodrago007@gmail.com; 3 Department of Dermatology and Venereology, Mother Teresa University Hospital Center, 1000 Tirana, Albania; hoxhallarigloria32@gmail.com

**Keywords:** skin infections, cutaneous diseases, infections of the mucous membranes

Dermatological practice frequently encounters infections of the skin and mucous membranes, ranging from primary viral and bacterial infections to the reactivation of latent viral pathogens. Notably, viral etiologies exhibit a complex relationship with clinical outcomes: a single virus may elicit different cutaneous and mucosal manifestations, just as a specific disease may arise from different viral triggers [1]. Some cutaneous viral and bacterial infections may not be merely localized conditions but can cause systemic complications through a vast clinical spectrum. Of particular concern are neurological complications, which arise from the complex interactions between direct infectious damage and secondary inflammatory pathways [1,2,3,4].

This Special Issue entitled “New Insights into Infectious Skin and Mucosal Diseases” of the Journal of Clinical Medicine sought to consolidate contemporary insights into the landscapes of cutaneous and mucosal infections from laboratory, clinical, epidemiological, and therapeutic perspectives (Table 1, Contributions 1–6).

Two articles in this issue explored the immunological aspects of some common viral infections: human papillomavirus (HPV) (Contribution 1) and human herpesviruses (HHV) 6 and 7 (Contribution 2).

The first study investigated the role of interleukins in the pathogenesis of warts, and the most common lesions caused by low-risk genotypes of HPV (LR-HPV).

Given its chronic course, LR-HPV infection, in particular when it involves the ano-genital sites, may impact the patient’s physical, psychological and sexual health and negatively influence their quality of life (QOL) [5]. Managing warts is therefore crucial for alleviating physical discomfort, preventing transmission, and halting lesion proliferation [6,7]. Most individuals with HPV mount an effective immune response and control the infection by generating a T helper (Th)1-dominated cell-mediated immune response, which is essential for an antiviral defense, primarily characterized by the production of interferon (IFN)-γ, tumor necrosis factor (TNF)-α, and interleukin (IL)-2 [8,9,10]. The disruption of the immune response and its shift from a Th1 to a Th2 response (characterized by the release of IL-4, IL-5, IL-6, IL-8, IL-10 and IL-31) are associated with altered virus recognition by the immune system, facilitating viral persistence for an indefinite period and resulting in the progression of lesions. About 10% of infected individuals develop a persistent infection, and they are at risk for progressive or recurrent disease [8,9,10]. Even in patients who are not immunosuppressed, LR-HPV benign lesions are difficult to eradicate; these patients can exhibit innate and adaptive local immunosuppression [8,9,10].

**Table 1:** Summary of the papers published in this Special Issue “New Insights into Infectious Skin and Mucosal Diseases” in the *Journal of Clinical Medicine*.

Contribution Number	Reference	Type of Publication	Number of Authors/Affiliations	Location of Authors’ Affiliation
1	Matei, C.; Diaconu, L.S.; Tampa, M.Interleukins in the Pathogenesis of Warts: Insight from the Last Decade. A Narrative Review. *J. Clin. Med.* **2025**, *14*, 2057 [11]	Review	3/4	Romania
2	Ciccarese, G.; Facciorusso, A.; Herzum, A.; Fidanzi, C.; Recalcati, S.; Foti, C.; Drago, F. Comparative Efficacy of Different Pharmacological Treatments for Pityriasis Rosea: A Network Meta-Analysis. *J. Clin. Med.* **2024**, *13*, 6666 [12]	Review	7/7	Italy
3	Bardazzi, F.; Sacchelli, L.; Clarizio, G.; Filippi, F.; Loi, C.; La Placa, M. Multiple-Site Lichen Planus: An Italian Case Series of 44 Patients. *J. Clin. Med.* **2025**, *14*, 7873 [13]	Article	6/2	Italy
4	Rychlik, K.; Sternicka-Rohde, J.; Nowicki, R.J.; Bieniaszewski, L.; Purzycka-Bohdan, D. Superficial Fungal Infections in Children—What Do We Know? *J. Clin. Med.* **2025**, *14*, 7380 [14]	Review	5/3	Poland
5	Addari, G.; Corbeddu, M.; Mugheddu, C.; Chessa, M.; Vivanet, G.; Ferreli, C.; Atzori, L. Tinea Capitis Induced by Barber Shaving: Isolation of Trichophyton tonsurans. *J. Clin. Med.* **2025**, *14*, 622 [15]	Article	7/2	Italy
6	Navarro-Pérez, D.; García-Oreja, S.; Álvaro-Afonso, F.J.; López-Moral, M.; Lázaro-Martínez, J.L.; Tardáguila-García, A. Red-Laser Photodynamic Therapy with Toluidine Blue Gel as an Adjuvant to Topical Antifungal Treatments for Onychomycosis in Patients with Diabetes: A Prospective Case Series. *J. Clin. Med.* **2025**, *14*, 1588 [16]	Article	6/2	Spain

Several immune evasion strategies may facilitate LR-HPV persistence, including a reduction in the number of local dendritic cells disrupting antigen presentation, altered cytokine activity, particularly suppression of the type I interferon pathway, and downregulation of adhesion molecules [9].

The intricate balance of multiple interleukins, carrying either pro- or anti-inflammatory properties and driving the immune reaction to HPV, is well-documented in HPV-induced cancerous lesions [17]. Conversely, data on non-malignant conditions such as warts are scarce. In their review, Matei C. et al. found that IL-17 was the most investigated IL, but studies have yielded conflicting results (Contribution 1). IL-17 is mainly produced by a subset of T CD4^+^ cells named T-helper 17 (Th-17) cells. IL-17 boosts the function of cytotoxic T lymphocytes, favoring HPV clearance. Some authors have shown higher serum levels of IL-17 in patients with warts, while others have found lower serum levels of the same cytokine among these patients compared to healthy controls. Two explanations have been proposed for these conflicting findings. The lower serum levels observed in patients with warts might stem from a longer disease duration, which leads to IL-17 depletion. Conversely, higher IL-17 concentrations could result from prolonged HPV infection triggering an inflammatory process that promotes the release of elevated amounts of this cytokine [18].

The serum level of different cytokines has also been investigated to elucidate the pathogenesis of pityriasis rosea (PR), an exanthematous disease associated with the endogenous systemic reactivation of HHV-6 and/or HHV-7. The upregulation of serum levels of IL-17, such as IL-22, IL-36, IFN-*γ*, vascular endothelial growth factor, CX3CL1/fractalkine, and CXCL10, has been detected in patients with PR compared to controls, representing further evidence that PR is associated with the activation of cellular immunity and induction of an inflammatory response [1]. Higher levels of circulating IL17A have been detected in the serum of patients with other viral systemic diseases like herpes zoster and COVID-19 compared to the controls [1].

Importantly, the role of Th17 cells in viral infections as well as IL-17 depends on the host’s genetic background, on the virus type, and on the Th17/Treg cell ratio [1,19,20,21]. Their role is generally considered harmful to the host since decreasing the production of IL2 and INF-γ, IL-17 can inhibit Th1 differentiation, prevent viral clearance, and cause inflammatory tissue damage. Conversely, in viral infections such as rotavirus and influenza virus, Th17 cells may play a defensive role contributing to viral clearance [1,19,20,21,22,23].

Concerning the clinical course, PR lesions are typically self-limiting; therefore, patient reassurance and rest are the preferred management strategy. However, atypical PR cases presenting with extensive, persistent lesions and systemic symptoms can impair the patient’s quality of life, justifying the prescription of active therapy [1]. Only twelve randomized controlled trials compared the efficacy of different pharmacological treatments for PR. The most recent network meta-analysis on this topic found that oral acyclovir was the best option in terms of a fast improvement and amelioration of systemic symptoms, while steroids and antihistamines seemed the best treatment for itch resolution (Contribution 2).

In the context of dermatological conditions linked to viral etiologies, oral lichen planus (OLP) may also be included, although there is still no conclusive evidence that HPV plays a role in its etiopathogenesis or in the malignant transformation of lesions [24].

A recent Italian study updated the clinical findings regarding the diverse manifestations and anatomical localizations of the multiple-site lichen planus, highlighting how oral lesions often represent the initial step of progressive cutaneous, mucosal and adnexal involvement (Contribution 3). Interestingly, most of the studied patients (88.6%) developed the disease in different body areas (mainly OLP, cutaneous lichen ruber planus and cutaneous/mucosal genital lichen planus) during the three-year follow-up period. Other patients (11.4%) developed five lichen subtypes (mainly OLP, cutaneous lichen ruber planus and cutaneous/mucosal genital lichen planus, frontal fibrosing alopecia and nail lichen planus). Given the complexity and dynamic nature of the condition, a multidisciplinary approach involving dermatologists, pathologists, dental clinicians, gynecologists, gastroenterologists and otorhinolaryngologists is recommended to tailor therapy and prevent complications.

Shifting the focus to fungal infections, highlighting their critical global emergence, the World Health Organization has recently issued a fungal priority pathogens list to guide and accelerate targeted research [25]. The pathogens were ranked and categorized into three priority groups (critical, high, and medium). The critical group includes *Cryptococcus neoformans*, *Candida auris*, *Aspergillus fumigatus* and *Candida albicans* [25]. These opportunistic agents can infect immunocompromised people, including those living with human immunodeficiency virus (HIV), hospitalized patients, organ transplant recipients and cancer patients undergoing chemotherapy, with a high mortality rate. Moreover, fungal pathogens are widely distributed in nature, often with increased resistance to current antifungal drugs [25,26].

Children are particularly susceptible to superficial fungal infections due to their immature immune systems. In recent years, the incidence of such infections in the pediatric population has increased, driven by a combination of predisposing factors. Underlying conditions encompass inherited and acquired immune deficiency, diabetes, and skin barrier dysfunctions such as atopic dermatitis. Prolonged warm and humid climates, low socioeconomic status, overcrowding, poor hygiene, and close contact with infected individuals or animals represent additional risk factors [26].

The most common superficial fungal infection in children is tinea capitis, mainly caused by the zoonotic pathogen *Microsporum canis* in central Europe (Contribution 4). Anthropophilic species causing scalp dermatophytoses such as *T. tonsurans*, which are more common in the United States of America, are increasingly reported across Europe, Israel, Argentina and Mexico. The rising cases in children and adults are attributed to multiple factors: (1) migration from endemic regions (West Africa, Latin America, or the Caribbean); (2) extensive administration of griseofulvin and emergence of drug-resistant strains due to mutations in the squalene epoxidase gene; (3) longer environmental persistence of *T. tonsurans* on fomites, such as hairbrushes, bedding, and caps, suggesting the possibility of indirect transmission in contexts such as barbershop and salon settings (Contribution 5); and (4) wide-variable clinical presentation that can mimic other dermatological conditions such as impetigo, psoriasis, hair tuft folliculitis and others (Contributions 4 and 5).

Terbinafine remains the preferred choice in the management of superficial dermatophyte infections in both children and adults due to its high efficacy and safety profile (Contributions 4 and 5).

Oral terbinafine is also the preferred agent for onychomycosis, particularly in severe cases (with involvement of the nail matrix in ≥50% of nails, and presence of dermatophytoma). However, like other antifungal drugs (itraconazole and fluconazole), it may be associated with systemic adverse events and interactions with other medications that have an inhibitory effect on the hepatic cytochrome P450 enzyme [27,28]. Consequently, terbinafine should be used with caution in patients with diabetes, immunosuppression, and those on several different drugs, even though these cohorts exhibit the highest susceptibility to fungal infections.

Antifungal topical therapy requires longer courses (often >48 weeks) and can be less effective than oral treatment owing to inadequate nail plate penetration [27,28]. Therefore, the combination of oral and topical antifungals may increase the cure rate.

Very recently, a novel strategy combining photodynamic therapy with topical antifungals has emerged in routine onychomycosis practice (Contribution 6). Photodynamic therapy initiates fungal apoptosis through exposure of the affected nails to specific light wavelengths that target cells exhibiting cellular accumulation of photosensitizers. Once activated, these agents generate cytotoxic reactive oxygen species, thereby mediating targeted fungal destruction [29].

More specifically, a regimen consisting of the application of topical Ciclopirox 8% once daily for 6 months supplemented with photodynamic therapy (three sessions every 2 weeks in the first 2 months) using toluidine blue gel as topical photosensitizer and a 635 nm diode laser (lasting 10 min) demonstrated clinical improvement and mycological cure without adverse events (Contribution 6). Therefore, the combination of these two modalities (a topical and a physical treatment) represents a promising therapeutic strategy for onychomycosis in cases of vulnerable patients who are contraindicated for oral therapies, particularly for the elderly, diabetic, or patients with multiple pathologies.

In conclusion, over the last decade, significant shifts in the epidemiology, virulence, and transmissibility of pathogens have been observed. Among viruses, both novel agents, such as Severe Acute Respiratory Syndrome Coronavirus 2 [1], and already well-known pathogens, like HPV and HHVs, have been linked to new cutaneous and mucosal challenges [1,10,24]. Among fungi, in recent years, in addition to the epidemiological shifts described for *Trichophyton tonsurans*, other dermatophytes have shown significant variations in geographic distribution. A rising number of cases of potentially sexually transmitted dermatophytosis have been reported in Europe, mainly associated with *Trichophyton (T.) mentagrophytes* genotype VII [30,31]. Moreover, dermatophytoses due to *T. indotineae*, originally confined to the Indian subcontinent, have been increasingly described in many countries worldwide, frequently attributed to travel and migration [32,33].

Future research should focus on preventing and managing both old and emerging fungal infections through innovative strategies, such as vaccines, antifungal peptides, nanotechnology, probiotics, and immunotherapy [34].

## Abbreviations

The following abbreviations are used in this manuscript:

HPV	Human papillomavirus
HHV	Human herpesvirus
OLP	Oral lichen planus
PR	Pityriasis rosea
HIV	Human immunodeficiency virus
IL	Interleukin
TNF	Tumor necrosis factor
INF	Interferon
QOL	Quality of life
